# Poly[tetra-*n*-butyl­ammonium [(μ_5_-benzene-1,3,5-tricarboxyl­ato)(μ_4_-benzene-1,3,5-tricarboxyl­ato)-μ_3_-hydroxido-trizincate] 0.25-hydrate]

**DOI:** 10.1107/S1600536811044485

**Published:** 2011-10-29

**Authors:** Xiao-Hong Zhu, Xiao-Chun Cheng

**Affiliations:** a Faculty of Life Science and Chemical Engineering, Huaiyin Institute of Technology, Huaian 223003, People’s Republic of China

## Abstract

In the asymmetric unit of title coordination polymer, {(C_16_H_36_N)[Zn_3_(C_9_H_3_O_6_)_2_(OH)]·0.25H_2_O}_*n*_, there are three independent Zn^2+^ cations, two benzene-1,3,5-tricarboxyl­ate ligands and a μ_3_-bridging hydroxide group, together with a tetra-*n*-butyl­ammonium counter-cation and a partially occupied water molecule of solvation (occupancy 0.25). Each Zn ion is coordinated by three carboxyl­ate O atoms and one O atom from the bridging hydroxide ion, displaying a slightly distorted tetra­hedral stereochemistry [overall Zn—O range = 1.875 (3)–1.987 (2) Å]. An intra­molecular hydrogen bond involving the hydroxide H atom and a carboxyl­ate O-atom acceptor is also present in the complex unit. The bridging benzene-1,3,5-tricarboxyl­ate anions generate a three-dimensional framework structure.

## Related literature

For a related structure, see: Su *et al.* (2009 ▶).
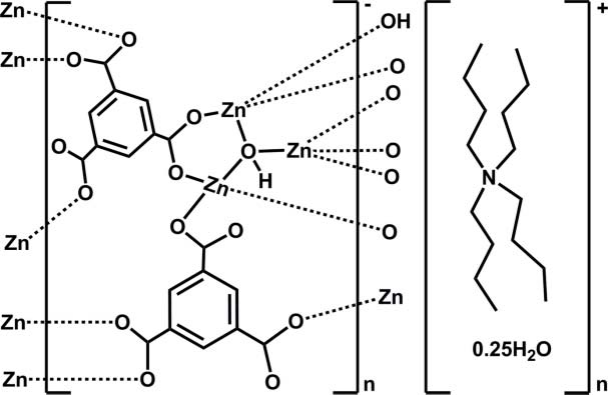


## Experimental

### 

#### Crystal data

(C_16_H_36_N)[Zn_3_(C_9_H_3_O_6_)_2_(OH)]·0.25H_2_O
*M* *_r_* = 873.86
Orthorhombic, 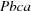

*a* = 16.295 (5) Å
*b* = 16.295 (5) Å
*c* = 28.946 (5) Å
*V* = 7686 (4) Å^3^
*Z* = 8
Mo *K*α radiation
μ = 1.92 mm^−1^
*T* = 293 K
0.20 × 0.20 × 0.18 mm

#### Data collection

Bruker APEXII CCD area-detector diffractometer
Absorption correction: multi-scan (*SADABS*; Sheldrick, 1996 ▶) *T*_min_ = 0.700, *T*_max_ = 0.724
46890 measured reflections
9492 independent reflections
6072 reflections with *I* > 2σ(*I*)
*R*_int_ = 0.060

#### Refinement

*R*[*F* ^2^ > 2σ(*F* ^2^)] = 0.037
*wR*(*F* ^2^) = 0.099
*S* = 0.93
9492 reflections
443 parameters
H-atom parameters constrained
Δρ_max_ = 1.24 e Å^−3^
Δρ_min_ = −1.35 e Å^−3^

### 

Data collection: *APEX2* (Bruker, 2008 ▶); cell refinement: *SAINT* (Bruker, 2008 ▶); data reduction: *SAINT*; program(s) used to solve structure: *SHELXS97* (Sheldrick, 2008 ▶); program(s) used to refine structure: *SHELXL97* (Sheldrick, 2008 ▶); molecular graphics: *DIAMOND* (Brandenburg, 2000 ▶); software used to prepare material for publication: *SHELXTL* (Sheldrick, 2008 ▶).

## Supplementary Material

Crystal structure: contains datablock(s) I, global. DOI: 10.1107/S1600536811044485/zs2153sup1.cif

Structure factors: contains datablock(s) I. DOI: 10.1107/S1600536811044485/zs2153Isup2.hkl

Supplementary material file. DOI: 10.1107/S1600536811044485/zs2153Isup4.cdx

Additional supplementary materials:  crystallographic information; 3D view; checkCIF report

## Figures and Tables

**Table 1:** Hydrogen-bond geometry (Å, °)

*D*—H⋯*A*	*D*—H	H⋯*A*	*D*⋯*A*	*D*—H⋯*A*
O13—H7⋯O8	0.93	1.73	2.609 (3)	158

**Fig. 1.**
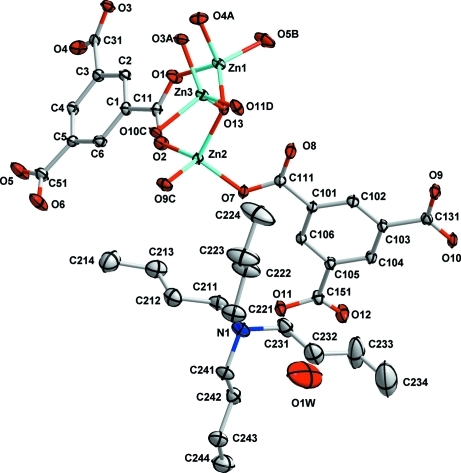
: The coordination environment of the Zn ions in the title complex together with the tetra-n-butylammonium counter-cation and the partial water molecule of solvation, with the ellipsoids drawn at the 30% probability level. The hydrogen atoms are omitted. Symmetry code: (A) x + 1/2, -y + 1/2, -z + 1; (B) -x + 3/2, y - 1/2, z; (C) -x + 2, y + 1/2, -z + 3/2; (D) x + 1/2, y, -z + 3/2.

**Fig. 2.**
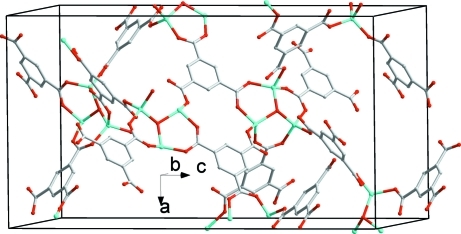
: The packing diagram of title coordination polymer complex. The tetra-n-butylammonium counter-cation and the partial water molecule of solvation are omitted for clarity.

## supplementary crystallographic information

### Comment

benzene-1,3,5-tricarboxylic acid is often used as organic ligand in the synthesis of metal complexes because of its abundant and variable coordination modes (Su *et al.*, 2009). Herein, we report the crystal structure of the title coordination polymer, [[(C_16_H_36_N) Zn_3_(C_9_H_3_O_3_)_2_(OH)].0.25(H_2_O)]_n_ (Fig. 1). In the asymmetric unit there are  three independent Zn cations, two benzene-1,3,5-tricarboxylate ligands and a µ_3_-bridging hydroxide group, together with a tetra-*n*-butylammonium counter-cation and a partial water molecule of solvation.  The coordination sphere about each ZnO_4_ centre comprises three carboxylate O atoms from separate benzene-1,3,5-tricarboxylate anions and one O atom from the bridging hydroxide anion, giving in each a slightly distorted tetrahedral stereochemistry [Zn—O ranges: 1.875 (3)–1.958 (2) Å (Zn1); 1.914 (2)–1.987 (2) Å (Zn2); 1.921 (2)–1.968 (2) Å (Zn3)]. An intramolecular hydrogen bond involving the hydroxo ligand H-donor and a carboxylate O-acceptor is also present in the complex unit (Table 1). The bridging benzene-1,3,5-tricarboxylate anions generate a three-dimensional framework structure (Fig. 2).

### Experimental

The reaction mixture of zinc nitrate hexahydrate (59.4 mg, 0.2 mmol), benzene-1,3,5-tricarboxylic acid (21.0 mg, 0.1 mmol), and 1 ml of aqueous tetra-*n*-butylammonium hydroxide solution (10%, *w*/*w*) in 12 ml of water was sealed in a 16 ml Teflon-lined stainless steel container and heated to 453 K for 3 days. After cooling to room temperature, colorless block crystals of the title complex were obtained.

### Refinement

The hydrogen atoms on all C atoms were located in geometrically idealized positions and constrained to ride on their parent atoms, with C—H = 0.93– 0.97 Å and *U*_iso_(H) = 1.2 or 1.5*U*_eq_(C). The hydrogen atom on the hydroxide group (O13) was found at a reasonable position in the difference-Fourier map and was constrained with *U*_iso_(H) = 1.2*U*_eq_(O)]. The partial water molecule of solvation (O1W) was refined with occupancy 0.25, while the attached hydrogen atoms could not be located.

### Crystal data

**Table:**

(C_16_H_36_N)[Zn_3_(C_9_H_3_O_6_)_2_(OH)]·0.25H_2_O	*F*(000) = 3600
*M**_r_* = 873.86	*D*_x_ = 1.510 Mg m^−^^3^
Orthorhombic, *P**b**c**a*	Mo *K*α radiation, λ = 0.71073 Å
Hall symbol: -P 2ac 2ab	Cell parameters from 6690 reflections
*a* = 16.295 (5) Å	θ = 2.3–25.4°
*b* = 16.295 (5) Å	µ = 1.92 mm^−^^1^
*c* = 28.946 (5) Å	*T* = 293 K
*V* = 7686 (4) Å^3^	Block, colorless
*Z* = 8	0.20 × 0.20 × 0.18 mm

### Data collection

**Table:**

Bruker APEXII CCD area-detector diffractometer	9492 independent reflections
Radiation source: fine-focus sealed tube	6072 reflections with *I* > 2σ(*I*)
graphite	*R*_int_ = 0.060
φ and ω scans	θ_max_ = 28.5°, θ_min_ = 1.9°
Absorption correction: multi-scan (*SADABS*; Sheldrick, 1996)	*h* = −21→16
*T*_min_ = 0.700, *T*_max_ = 0.724	*k* = −16→21
46890 measured reflections	*l* = −38→37

### Refinement

**Table:**

Refinement on *F*^2^	Primary atom site location: structure-invariant direct methods
Least-squares matrix: full	Secondary atom site location: difference Fourier map
*R*[*F*^2^ > 2σ(*F*^2^)] = 0.037	Hydrogen site location: inferred from neighbouring sites
*wR*(*F*^2^) = 0.099	H-atom parameters constrained
*S* = 0.93	*w* = 1/[σ^2^(*F*_o_^2^) + (0.040*P*)^2^] where *P* = (*F*_o_^2^ + 2*F*_c_^2^)/3
9492 reflections	(Δ/σ)_max_ = 0.002
443 parameters	Δρ_max_ = 1.24 e Å^−^^3^
0 restraints	Δρ_min_ = −1.35 e Å^−^^3^

### Special details

**Table:**

Geometry. All esds (except the esd in the dihedral angle between two l.s. planes) are estimated using the full covariance matrix. The cell esds are taken into account individually in the estimation of esds in distances, angles and torsion angles; correlations between esds in cell parameters are only used when they are defined by crystal symmetry. An approximate (isotropic) treatment of cell esds is used for estimating esds involving l.s. planes.
Refinement. Refinement of *F*^2^ against ALL reflections. The weighted *R*-factor *wR* and goodness of fit *S* are based on *F*^2^, conventional *R*-factors *R* are based on *F*, with *F* set to zero for negative *F*^2^. The threshold expression of *F*^2^ > σ(*F*^2^) is used only for calculating *R*-factors(gt) *etc*. and is not relevant to the choice of reflections for refinement. *R*-factors based on *F*^2^ are statistically about twice as large as those based on *F*, and *R*- factors based on ALL data will be even larger.

### Fractional atomic coordinates and isotropic or equivalent isotropic displacement parameters (Å^2^)

**Table:**

	*x*	*y*	*z*	*U*_iso_*/*U*_eq_	Occ. (<1)
C1	0.76518 (19)	0.3324 (2)	0.57153 (11)	0.0241 (8)	
C2	0.72626 (19)	0.2998 (2)	0.53324 (11)	0.0250 (8)	
H1	0.7475	0.2534	0.5188	0.030*	
C3	0.65577 (19)	0.3364 (2)	0.51636 (11)	0.0224 (7)	
C4	0.62623 (19)	0.4065 (2)	0.53724 (11)	0.0235 (7)	
H2	0.5783	0.4305	0.5262	0.028*	
C5	0.6665 (2)	0.4418 (2)	0.57412 (11)	0.0240 (8)	
C6	0.7354 (2)	0.4038 (2)	0.59158 (11)	0.0265 (8)	
H3	0.7621	0.4262	0.6170	0.032*	
C11	0.8392 (2)	0.2900 (2)	0.59169 (12)	0.0252 (8)	
C31	0.6116 (2)	0.3057 (2)	0.47464 (11)	0.0230 (7)	
C51	0.6355 (2)	0.5218 (2)	0.59296 (13)	0.0332 (9)	
C101	0.9004 (2)	0.0957 (2)	0.76805 (11)	0.0231 (7)	
C102	0.92903 (19)	0.0206 (2)	0.78223 (11)	0.0247 (8)	
H4	0.9737	−0.0029	0.7672	0.030*	
C103	0.89223 (19)	−0.0204 (2)	0.81857 (11)	0.0225 (7)	
C104	0.82612 (19)	0.0157 (2)	0.84119 (11)	0.0256 (8)	
H5	0.8012	−0.0113	0.8658	0.031*	
C105	0.79740 (19)	0.0912 (2)	0.82739 (11)	0.0244 (8)	
C106	0.83441 (19)	0.1305 (2)	0.79074 (11)	0.0240 (8)	
H6	0.8146	0.1813	0.7811	0.029*	
C111	0.9409 (2)	0.1396 (2)	0.72901 (12)	0.0274 (8)	
C131	0.9225 (2)	−0.1027 (2)	0.83342 (11)	0.0246 (8)	
C151	0.7268 (2)	0.1304 (2)	0.85213 (12)	0.0279 (8)	
C211	0.8723 (2)	0.3453 (3)	0.82377 (14)	0.0455 (11)	
H9	0.8856	0.3017	0.8022	0.055*	
H8	0.8161	0.3366	0.8337	0.055*	
C212	0.8758 (3)	0.4257 (3)	0.79796 (15)	0.0527 (12)	
H10	0.8596	0.4701	0.8183	0.063*	
H11	0.9316	0.4360	0.7878	0.063*	
C213	0.81928 (19)	0.4232 (2)	0.75671 (12)	0.0639 (14)	
H13	0.7634	0.4147	0.7672	0.077*	
H12	0.8342	0.3772	0.7371	0.077*	
C214	0.82376 (19)	0.5032 (2)	0.72833 (12)	0.0888 (19)	
H14	0.8231	0.5494	0.7489	0.133*	
H16	0.7775	0.5062	0.7079	0.133*	
H15	0.8735	0.5038	0.7106	0.133*	
C221	1.0164 (2)	0.3513 (3)	0.85266 (15)	0.0587 (14)	
H18	1.0504	0.3435	0.8798	0.070*	
H17	1.0220	0.4082	0.8432	0.070*	
C222	1.0494 (3)	0.2962 (4)	0.81374 (18)	0.0782 (17)	
H19	1.0380	0.2393	0.8212	0.094*	
H20	1.0209	0.3092	0.7853	0.094*	
C223	1.1379 (2)	0.3062 (3)	0.80653 (17)	0.108 (2)	
H22	1.1662	0.2968	0.8356	0.129*	
H21	1.1489	0.3622	0.7970	0.129*	
C224	1.1714 (2)	0.2487 (3)	0.77085 (17)	0.137 (3)	
H25	1.1587	0.1932	0.7793	0.205*	
H24	1.2299	0.2552	0.7689	0.205*	
H23	1.1472	0.2609	0.7414	0.205*	
C231	0.9169 (3)	0.2499 (3)	0.88305 (16)	0.0545 (12)	
H27	0.9338	0.2125	0.8588	0.065*	
H26	0.8589	0.2408	0.8886	0.065*	
C232	0.9637 (3)	0.2281 (3)	0.92683 (17)	0.0724 (16)	
H28	1.0222	0.2279	0.9208	0.087*	
H29	0.9525	0.2683	0.9507	0.087*	
C233	0.9361 (3)	0.1441 (3)	0.94246 (19)	0.132	
H30	0.9478	0.1044	0.9184	0.158*	
H31	0.8772	0.1446	0.9475	0.158*	
C234	0.9790 (3)	0.1180 (3)	0.98675 (19)	0.172 (4)	
H32	1.0347	0.1371	0.9864	0.258*	
H33	0.9785	0.0592	0.9891	0.258*	
H34	0.9508	0.1411	1.0128	0.258*	
C241	0.9052 (2)	0.3982 (3)	0.90251 (14)	0.0472 (11)	
H35	0.9461	0.3962	0.9268	0.057*	
H36	0.9072	0.4526	0.8889	0.057*	
C242	0.8221 (2)	0.3864 (3)	0.92395 (14)	0.0449 (11)	
H38	0.8185	0.3318	0.9372	0.054*	
H37	0.7800	0.3913	0.9004	0.054*	
C243	0.8077 (2)	0.4499 (2)	0.96125 (11)	0.0593 (13)	
H39	0.8159	0.5041	0.9482	0.071*	
H40	0.8481	0.4423	0.9855	0.071*	
C244	0.7220 (2)	0.4457 (2)	0.98250 (11)	0.0803 (17)	
H42	0.6815	0.4491	0.9585	0.120*	
H43	0.7148	0.4906	1.0036	0.120*	
H41	0.7158	0.3948	0.9988	0.120*	
N1	0.92799 (19)	0.3363 (2)	0.86590 (11)	0.0427 (9)	
O1	0.87184 (14)	0.23552 (16)	0.56768 (8)	0.0362 (6)	
O2	0.86264 (14)	0.31397 (16)	0.63058 (8)	0.0355 (6)	
O3	0.64976 (14)	0.25944 (15)	0.44788 (8)	0.0305 (6)	
O4	0.53918 (13)	0.32934 (16)	0.46956 (8)	0.0337 (6)	
O5	0.57056 (17)	0.54736 (18)	0.57447 (10)	0.0531 (6)	
O6	0.67374 (17)	0.55794 (17)	0.62306 (10)	0.0531 (6)	
O7	0.91524 (14)	0.21258 (14)	0.72211 (8)	0.0295 (6)	
O8	0.99505 (16)	0.10514 (16)	0.70684 (9)	0.0431 (7)	
O9	0.98565 (14)	−0.12995 (15)	0.81338 (8)	0.0315 (6)	
O10	0.88317 (14)	−0.13893 (14)	0.86430 (8)	0.0316 (6)	
O11	0.71941 (14)	0.20737 (15)	0.84644 (8)	0.0329 (6)	
O12	0.68018 (16)	0.09061 (16)	0.87645 (9)	0.0468 (8)	
O13	1.02002 (12)	0.19730 (13)	0.63449 (7)	0.0184 (5)	
H7	1.0246	0.1607	0.6588	0.022*	
Zn1	0.95951 (2)	0.15787 (2)	0.580582 (13)	0.02168 (10)	
Zn2	0.95001 (2)	0.27515 (2)	0.669671 (12)	0.01930 (10)	
Zn3	1.12713 (2)	0.24495 (2)	0.618089 (13)	0.02092 (10)	
O1W	0.1549 (8)	0.1219 (9)	0.8850 (4)	0.083	0.25

### Atomic displacement parameters (Å^2^)

**Table:**

	*U*^11^	*U*^22^	*U*^33^	*U*^12^	*U*^13^	*U*^23^
C1	0.0221 (17)	0.025 (2)	0.0250 (18)	0.0056 (15)	−0.0026 (14)	0.0001 (15)
C2	0.0276 (19)	0.024 (2)	0.0238 (18)	0.0059 (15)	−0.0031 (15)	−0.0047 (15)
C3	0.0214 (17)	0.0221 (19)	0.0237 (18)	0.0008 (14)	−0.0029 (14)	−0.0010 (15)
C4	0.0193 (17)	0.025 (2)	0.0261 (18)	0.0038 (15)	−0.0005 (14)	0.0023 (15)
C5	0.0257 (18)	0.024 (2)	0.0222 (18)	0.0044 (15)	−0.0021 (14)	−0.0034 (15)
C6	0.0288 (19)	0.029 (2)	0.0223 (18)	0.0036 (16)	−0.0047 (15)	−0.0051 (16)
C11	0.0239 (18)	0.025 (2)	0.0265 (19)	0.0037 (15)	−0.0032 (15)	0.0027 (16)
C31	0.0271 (19)	0.0205 (19)	0.0212 (18)	−0.0002 (15)	−0.0043 (14)	0.0029 (15)
C51	0.039 (2)	0.025 (2)	0.036 (2)	0.0037 (17)	−0.0047 (17)	−0.0082 (17)
C101	0.0270 (18)	0.0190 (19)	0.0233 (18)	0.0021 (14)	0.0033 (14)	0.0050 (15)
C102	0.0234 (18)	0.024 (2)	0.0265 (19)	0.0051 (15)	0.0067 (14)	0.0038 (15)
C103	0.0229 (18)	0.0184 (19)	0.0262 (19)	0.0025 (14)	0.0007 (14)	0.0027 (15)
C104	0.0266 (19)	0.022 (2)	0.028 (2)	0.0014 (15)	0.0049 (15)	0.0054 (15)
C105	0.0234 (18)	0.022 (2)	0.0283 (19)	0.0026 (14)	0.0033 (14)	0.0021 (16)
C106	0.0269 (18)	0.0178 (19)	0.0272 (19)	0.0031 (14)	0.0007 (15)	0.0044 (15)
C111	0.030 (2)	0.023 (2)	0.029 (2)	0.0045 (15)	0.0035 (16)	0.0059 (16)
C131	0.0287 (19)	0.0195 (19)	0.0256 (19)	−0.0021 (15)	−0.0025 (15)	0.0001 (15)
C151	0.0264 (19)	0.024 (2)	0.033 (2)	0.0042 (16)	0.0007 (16)	0.0012 (17)
C211	0.041 (2)	0.049 (3)	0.047 (3)	−0.009 (2)	−0.005 (2)	−0.014 (2)
C212	0.047 (3)	0.052 (3)	0.059 (3)	−0.008 (2)	0.004 (2)	−0.005 (2)
C213	0.058 (3)	0.073 (4)	0.061 (3)	0.006 (3)	−0.001 (3)	−0.013 (3)
C214	0.091 (4)	0.103 (5)	0.072 (4)	0.023 (4)	0.002 (3)	0.019 (4)
C221	0.037 (3)	0.076 (4)	0.064 (3)	−0.011 (2)	−0.005 (2)	−0.023 (3)
C222	0.049 (3)	0.099 (5)	0.087 (4)	−0.002 (3)	0.003 (3)	−0.042 (4)
C223	0.062 (4)	0.137 (7)	0.124 (5)	−0.019 (4)	0.031 (4)	−0.057 (5)
C224	0.101 (5)	0.134 (7)	0.175 (7)	−0.015 (5)	0.060 (5)	−0.084 (6)
C231	0.060 (3)	0.039 (3)	0.064 (3)	−0.002 (2)	−0.015 (2)	−0.010 (2)
C232	0.079 (4)	0.052 (3)	0.087 (4)	0.003 (3)	−0.035 (3)	−0.011 (3)
C233	0.202	0.067	0.127	0.015	−0.102	−0.027
C234	0.229 (9)	0.096 (7)	0.191 (9)	0.001 (6)	−0.073 (7)	0.030 (6)
C241	0.055 (3)	0.042 (3)	0.044 (3)	−0.008 (2)	−0.008 (2)	−0.014 (2)
C242	0.047 (3)	0.040 (3)	0.048 (3)	0.001 (2)	−0.004 (2)	−0.004 (2)
C243	0.078 (3)	0.054 (3)	0.045 (3)	−0.005 (3)	−0.002 (2)	−0.009 (2)
C244	0.088 (4)	0.081 (5)	0.072 (4)	0.007 (3)	0.017 (3)	−0.017 (3)
N1	0.0382 (19)	0.041 (2)	0.049 (2)	−0.0068 (16)	−0.0052 (16)	−0.0132 (18)
O1	0.0314 (14)	0.0412 (17)	0.0361 (14)	0.0195 (12)	−0.0088 (11)	−0.0106 (13)
O2	0.0377 (15)	0.0389 (17)	0.0298 (14)	0.0149 (12)	−0.0170 (11)	−0.0074 (12)
O3	0.0377 (14)	0.0286 (15)	0.0252 (13)	0.0103 (11)	−0.0107 (11)	−0.0093 (11)
O4	0.0231 (13)	0.0478 (18)	0.0303 (14)	0.0050 (12)	−0.0091 (11)	−0.0062 (12)
O5	0.0554 (13)	0.0365 (13)	0.0674 (14)	0.0197 (10)	−0.0221 (11)	−0.0241 (11)
O6	0.0554 (13)	0.0365 (13)	0.0674 (14)	0.0197 (10)	−0.0221 (11)	−0.0241 (11)
O7	0.0387 (14)	0.0203 (14)	0.0295 (14)	0.0043 (11)	0.0108 (11)	0.0088 (11)
O8	0.0517 (17)	0.0348 (17)	0.0428 (16)	0.0208 (13)	0.0261 (13)	0.0201 (13)
O9	0.0312 (14)	0.0268 (15)	0.0367 (15)	0.0121 (11)	0.0079 (11)	0.0098 (12)
O10	0.0410 (15)	0.0190 (14)	0.0348 (14)	0.0014 (11)	0.0127 (12)	0.0104 (11)
O11	0.0316 (14)	0.0227 (15)	0.0445 (16)	0.0056 (11)	0.0121 (11)	0.0060 (12)
O12	0.0440 (16)	0.0316 (17)	0.065 (2)	0.0041 (13)	0.0315 (14)	0.0127 (15)
O13	0.0187 (11)	0.0167 (12)	0.0199 (11)	0.0021 (9)	−0.0016 (9)	0.0023 (10)
Zn1	0.01888 (19)	0.0196 (2)	0.0266 (2)	−0.00071 (16)	0.00158 (16)	−0.00610 (17)
Zn2	0.02036 (19)	0.0173 (2)	0.0202 (2)	0.00008 (16)	−0.00066 (15)	0.00052 (16)
Zn3	0.01865 (19)	0.0210 (2)	0.0231 (2)	0.00059 (16)	−0.00087 (16)	−0.00463 (17)
O1W	0.083	0.087	0.079	0.064	−0.013	−0.012

### Geometric parameters (Å, °)

**Table:**

C1—C2	1.383 (4)	C222—H19	0.9700
C1—C6	1.388 (4)	C222—H20	0.9700
C1—C11	1.507 (4)	C223—C224	1.4970
C2—C3	1.383 (4)	C223—H22	0.9700
C2—H1	0.9300	C223—H21	0.9700
C3—C4	1.380 (4)	C224—H25	0.9600
C3—C31	1.492 (4)	C224—H24	0.9600
C4—C5	1.378 (4)	C224—H23	0.9600
C4—H2	0.9300	C231—N1	1.502 (5)
C5—C6	1.378 (4)	C231—C232	1.521 (6)
C5—C51	1.501 (5)	C231—H27	0.9700
C6—H3	0.9300	C231—H26	0.9700
C11—O1	1.247 (4)	C232—C233	1.511 (7)
C11—O2	1.251 (4)	C232—H28	0.9700
C31—O3	1.247 (4)	C232—H29	0.9700
C31—O4	1.251 (4)	C233—C234	1.5206
C51—O6	1.222 (4)	C233—H30	0.9700
C51—O5	1.257 (4)	C233—H31	0.9700
C101—C102	1.373 (4)	C234—H32	0.9600
C101—C106	1.382 (4)	C234—H33	0.9600
C101—C111	1.491 (4)	C234—H34	0.9600
C102—C103	1.383 (4)	C241—C242	1.502 (5)
C102—H4	0.9300	C241—N1	1.510 (5)
C103—C104	1.391 (4)	C241—H35	0.9700
C103—C131	1.492 (5)	C241—H36	0.9700
C104—C105	1.375 (4)	C242—C243	1.514 (5)
C104—H5	0.9300	C242—H38	0.9700
C105—C106	1.378 (4)	C242—H37	0.9700
C105—C151	1.498 (4)	C243—C244	1.5272
C106—H6	0.9300	C243—H39	0.9700
C111—O8	1.227 (4)	C243—H40	0.9700
C111—O7	1.277 (4)	C244—H42	0.9600
C131—O10	1.248 (4)	C244—H43	0.9600
C131—O9	1.262 (4)	C244—H41	0.9600
C151—O12	1.221 (4)	O1—Zn1	1.945 (2)
C151—O11	1.271 (4)	O2—Zn2	1.926 (2)
C211—C212	1.510 (6)	O3—Zn3^i^	1.946 (2)
C211—N1	1.527 (5)	O4—Zn1^i^	1.958 (2)
C211—H9	0.9700	O5—Zn1^ii^	1.875 (3)
C211—H8	0.9700	O7—Zn2	1.914 (2)
C212—C213	1.509 (5)	O9—Zn2^iii^	1.932 (2)
C212—H10	0.9700	O10—Zn3^iii^	1.967 (2)
C212—H11	0.9700	O11—Zn3^iv^	1.921 (2)
C213—C214	1.5431	O13—Zn1	1.954 (2)
C213—H13	0.9700	O13—Zn3	1.968 (2)
C213—H12	0.9700	O13—Zn2	1.987 (2)
C214—H14	0.9600	O13—H7	0.9259
C214—H16	0.9600	Zn1—O5^v^	1.875 (3)
C214—H15	0.9600	Zn1—O4^vi^	1.958 (2)
C221—N1	1.511 (5)	Zn2—O9^vii^	1.932 (2)
C221—C222	1.538 (6)	Zn3—O11^viii^	1.921 (2)
C221—H18	0.9700	Zn3—O3^vi^	1.946 (2)
C221—H17	0.9700	Zn3—O10^vii^	1.967 (2)
C222—C223	1.466 (5)		
			
C2—C1—C6	119.7 (3)	H22—C223—H21	107.8
C2—C1—C11	120.1 (3)	C223—C224—H25	109.5
C6—C1—C11	120.1 (3)	C223—C224—H24	109.5
C1—C2—C3	120.0 (3)	H25—C224—H24	109.5
C1—C2—H1	120.0	C223—C224—H23	109.5
C3—C2—H1	120.0	H25—C224—H23	109.5
C4—C3—C2	119.4 (3)	H24—C224—H23	109.5
C4—C3—C31	117.6 (3)	N1—C231—C232	115.7 (4)
C2—C3—C31	122.9 (3)	N1—C231—H27	108.3
C5—C4—C3	121.2 (3)	C232—C231—H27	108.3
C5—C4—H2	119.4	N1—C231—H26	108.3
C3—C4—H2	119.4	C232—C231—H26	108.3
C6—C5—C4	119.0 (3)	H27—C231—H26	107.4
C6—C5—C51	122.1 (3)	C233—C232—C231	108.2 (4)
C4—C5—C51	118.9 (3)	C233—C232—H28	110.1
C5—C6—C1	120.5 (3)	C231—C232—H28	110.1
C5—C6—H3	119.7	C233—C232—H29	110.1
C1—C6—H3	119.7	C231—C232—H29	110.1
O1—C11—O2	126.4 (3)	H28—C232—H29	108.4
O1—C11—C1	116.9 (3)	C232—C233—C234	111.7 (3)
O2—C11—C1	116.7 (3)	C232—C233—H30	109.3
O3—C31—O4	125.7 (3)	C234—C233—H30	109.3
O3—C31—C3	117.7 (3)	C232—C233—H31	109.3
O4—C31—C3	116.6 (3)	C234—C233—H31	109.3
O6—C51—O5	125.0 (4)	H30—C233—H31	107.9
O6—C51—C5	120.4 (3)	C233—C234—H32	109.5
O5—C51—C5	114.6 (3)	C233—C234—H33	109.5
C102—C101—C106	119.2 (3)	H32—C234—H33	109.5
C102—C101—C111	120.2 (3)	C233—C234—H34	109.5
C106—C101—C111	120.6 (3)	H32—C234—H34	109.5
C101—C102—C103	120.7 (3)	H33—C234—H34	109.5
C101—C102—H4	119.6	C242—C241—N1	115.2 (3)
C103—C102—H4	119.6	C242—C241—H35	108.5
C102—C103—C104	119.3 (3)	N1—C241—H35	108.5
C102—C103—C131	120.7 (3)	C242—C241—H36	108.5
C104—C103—C131	120.0 (3)	N1—C241—H36	108.5
C105—C104—C103	120.3 (3)	H35—C241—H36	107.5
C105—C104—H5	119.8	C241—C242—C243	110.3 (3)
C103—C104—H5	119.8	C241—C242—H38	109.6
C104—C105—C106	119.4 (3)	C243—C242—H38	109.6
C104—C105—C151	120.2 (3)	C241—C242—H37	109.6
C106—C105—C151	120.4 (3)	C243—C242—H37	109.6
C105—C106—C101	121.1 (3)	H38—C242—H37	108.1
C105—C106—H6	119.5	C242—C243—C244	113.5 (2)
C101—C106—H6	119.5	C242—C243—H39	108.9
O8—C111—O7	125.4 (3)	C244—C243—H39	108.9
O8—C111—C101	119.7 (3)	C242—C243—H40	108.9
O7—C111—C101	114.8 (3)	C244—C243—H40	108.9
O10—C131—O9	125.5 (3)	H39—C243—H40	107.7
O10—C131—C103	117.5 (3)	C243—C244—H42	109.5
O9—C131—C103	116.9 (3)	C243—C244—H43	109.5
O12—C151—O11	122.6 (3)	H42—C244—H43	109.5
O12—C151—C105	121.8 (3)	C243—C244—H41	109.5
O11—C151—C105	115.5 (3)	H42—C244—H41	109.5
C212—C211—N1	117.1 (3)	H43—C244—H41	109.5
C212—C211—H9	108.0	C231—N1—C241	111.4 (3)
N1—C211—H9	108.0	C231—N1—C221	110.5 (3)
C212—C211—H8	108.0	C241—N1—C221	107.7 (3)
N1—C211—H8	108.0	C231—N1—C211	106.4 (3)
H9—C211—H8	107.3	C241—N1—C211	110.5 (3)
C213—C212—C211	110.2 (3)	C221—N1—C211	110.4 (3)
C213—C212—H10	109.6	C11—O1—Zn1	132.1 (2)
C211—C212—H10	109.6	C11—O2—Zn2	130.7 (2)
C213—C212—H11	109.6	C31—O3—Zn3^i^	122.5 (2)
C211—C212—H11	109.6	C31—O4—Zn1^i^	138.2 (2)
H10—C212—H11	108.1	C51—O5—Zn1^ii^	119.9 (2)
C212—C213—C214	111.7 (2)	C111—O7—Zn2	121.6 (2)
C212—C213—H13	109.3	C131—O9—Zn2^iii^	127.4 (2)
C214—C213—H13	109.3	C131—O10—Zn3^iii^	133.2 (2)
C212—C213—H12	109.3	C151—O11—Zn3^iv^	108.6 (2)
C214—C213—H12	109.3	Zn1—O13—Zn3	112.61 (10)
H13—C213—H12	107.9	Zn1—O13—Zn2	109.24 (9)
C213—C214—H14	109.5	Zn3—O13—Zn2	112.38 (10)
C213—C214—H16	109.5	Zn1—O13—H7	115.8
H14—C214—H16	109.5	Zn3—O13—H7	111.5
C213—C214—H15	109.5	Zn2—O13—H7	93.9
H14—C214—H15	109.5	O5^v^—Zn1—O1	114.51 (12)
H16—C214—H15	109.5	O5^v^—Zn1—O13	121.54 (11)
N1—C221—C222	115.1 (3)	O1—Zn1—O13	108.06 (10)
N1—C221—H18	108.5	O5^v^—Zn1—O4^vi^	101.86 (11)
C222—C221—H18	108.5	O1—Zn1—O4^vi^	105.99 (11)
N1—C221—H17	108.5	O13—Zn1—O4^vi^	102.85 (9)
C222—C221—H17	108.5	O7—Zn2—O2	114.97 (11)
H18—C221—H17	107.5	O7—Zn2—O9^vii^	112.68 (10)
C223—C222—C221	112.6 (4)	O2—Zn2—O9^vii^	106.72 (11)
C223—C222—H19	109.1	O7—Zn2—O13	103.67 (9)
C221—C222—H19	109.1	O2—Zn2—O13	109.45 (9)
C223—C222—H20	109.1	O9^vii^—Zn2—O13	109.25 (9)
C221—C222—H20	109.1	O11^viii^—Zn3—O3^vi^	111.37 (10)
H19—C222—H20	107.8	O11^viii^—Zn3—O10^vii^	103.59 (10)
C222—C223—C224	112.8 (3)	O3^vi^—Zn3—O10^vii^	107.81 (10)
C222—C223—H22	109.0	O11^viii^—Zn3—O13	116.07 (10)
C224—C223—H22	109.0	O3^vi^—Zn3—O13	112.96 (9)
C222—C223—H21	109.0	O10^vii^—Zn3—O13	103.96 (9)
C224—C223—H21	109.0		
			
C6—C1—C2—C3	−2.9 (5)	C232—C231—N1—C221	63.2 (5)
C11—C1—C2—C3	176.5 (3)	C232—C231—N1—C211	−176.9 (4)
C1—C2—C3—C4	1.8 (5)	C242—C241—N1—C231	−51.9 (5)
C1—C2—C3—C31	178.7 (3)	C242—C241—N1—C221	−173.2 (4)
C2—C3—C4—C5	1.2 (5)	C242—C241—N1—C211	66.2 (5)
C31—C3—C4—C5	−175.8 (3)	C222—C221—N1—C231	61.0 (5)
C3—C4—C5—C6	−3.0 (5)	C222—C221—N1—C241	−177.2 (4)
C3—C4—C5—C51	175.0 (3)	C222—C221—N1—C211	−56.5 (5)
C4—C5—C6—C1	1.8 (5)	C212—C211—N1—C231	−176.3 (4)
C51—C5—C6—C1	−176.1 (3)	C212—C211—N1—C241	62.6 (5)
C2—C1—C6—C5	1.1 (5)	C212—C211—N1—C221	−56.4 (5)
C11—C1—C6—C5	−178.3 (3)	O2—C11—O1—Zn1	8.2 (6)
C2—C1—C11—O1	13.4 (5)	C1—C11—O1—Zn1	−173.1 (2)
C6—C1—C11—O1	−167.2 (3)	O1—C11—O2—Zn2	−4.1 (6)
C2—C1—C11—O2	−167.9 (3)	C1—C11—O2—Zn2	177.2 (2)
C6—C1—C11—O2	11.5 (5)	O4—C31—O3—Zn3^i^	29.4 (5)
C4—C3—C31—O3	158.8 (3)	C3—C31—O3—Zn3^i^	−150.2 (2)
C2—C3—C31—O3	−18.1 (5)	O3—C31—O4—Zn1^i^	−22.1 (6)
C4—C3—C31—O4	−20.8 (5)	C3—C31—O4—Zn1^i^	157.5 (3)
C2—C3—C31—O4	162.3 (3)	O6—C51—O5—Zn1^ii^	16.2 (6)
C6—C5—C51—O6	4.0 (6)	C5—C51—O5—Zn1^ii^	−161.9 (2)
C4—C5—C51—O6	−173.9 (3)	O8—C111—O7—Zn2	8.0 (5)
C6—C5—C51—O5	−177.8 (3)	C101—C111—O7—Zn2	−172.9 (2)
C4—C5—C51—O5	4.3 (5)	O10—C131—O9—Zn2^iii^	−10.7 (5)
C106—C101—C102—C103	0.5 (5)	C103—C131—O9—Zn2^iii^	169.7 (2)
C111—C101—C102—C103	179.7 (3)	O9—C131—O10—Zn3^iii^	−22.6 (5)
C101—C102—C103—C104	−0.8 (5)	C103—C131—O10—Zn3^iii^	156.9 (2)
C101—C102—C103—C131	178.9 (3)	O12—C151—O11—Zn3^iv^	0.4 (4)
C102—C103—C104—C105	0.4 (5)	C105—C151—O11—Zn3^iv^	−178.8 (2)
C131—C103—C104—C105	−179.4 (3)	C11—O1—Zn1—O5^v^	120.9 (3)
C103—C104—C105—C106	0.4 (5)	C11—O1—Zn1—O13	−18.0 (4)
C103—C104—C105—C151	−179.4 (3)	C11—O1—Zn1—O4^vi^	−127.6 (3)
C104—C105—C106—C101	−0.8 (5)	Zn3—O13—Zn1—O5^v^	119.45 (13)
C151—C105—C106—C101	179.0 (3)	Zn2—O13—Zn1—O5^v^	−114.94 (13)
C102—C101—C106—C105	0.3 (5)	Zn3—O13—Zn1—O1	−105.14 (12)
C111—C101—C106—C105	−178.9 (3)	Zn2—O13—Zn1—O1	20.47 (13)
C102—C101—C111—O8	6.6 (5)	Zn3—O13—Zn1—O4^vi^	6.67 (13)
C106—C101—C111—O8	−174.2 (3)	Zn2—O13—Zn1—O4^vi^	132.28 (11)
C102—C101—C111—O7	−172.5 (3)	C111—O7—Zn2—O2	122.9 (3)
C106—C101—C111—O7	6.7 (5)	C111—O7—Zn2—O9^vii^	−114.6 (3)
C102—C103—C131—O10	−175.2 (3)	C111—O7—Zn2—O13	3.4 (3)
C104—C103—C131—O10	4.5 (5)	C11—O2—Zn2—O7	−105.3 (3)
C102—C103—C131—O9	4.4 (5)	C11—O2—Zn2—O9^vii^	129.0 (3)
C104—C103—C131—O9	−175.9 (3)	C11—O2—Zn2—O13	10.9 (3)
C104—C105—C151—O12	−18.6 (5)	Zn1—O13—Zn2—O7	104.57 (11)
C106—C105—C151—O12	161.6 (3)	Zn3—O13—Zn2—O7	−129.69 (11)
C104—C105—C151—O11	160.6 (3)	Zn1—O13—Zn2—O2	−18.56 (14)
C106—C105—C151—O11	−19.1 (5)	Zn3—O13—Zn2—O2	107.18 (12)
N1—C211—C212—C213	178.2 (3)	Zn1—O13—Zn2—O9^vii^	−135.08 (11)
C211—C212—C213—C214	−177.8 (2)	Zn3—O13—Zn2—O9^vii^	−9.34 (13)
N1—C221—C222—C223	−172.6 (4)	Zn1—O13—Zn3—O11^viii^	−131.35 (11)
C221—C222—C223—C224	176.2 (3)	Zn2—O13—Zn3—O11^viii^	104.76 (12)
N1—C231—C232—C233	172.1 (4)	Zn1—O13—Zn3—O3^vi^	−0.97 (14)
C231—C232—C233—C234	−179.0 (3)	Zn2—O13—Zn3—O3^vi^	−124.86 (11)
N1—C241—C242—C243	177.8 (3)	Zn1—O13—Zn3—O10^vii^	115.62 (11)
C241—C242—C243—C244	176.0 (2)	Zn2—O13—Zn3—O10^vii^	−8.27 (12)
C232—C231—N1—C241	−56.5 (5)		

Symmetry codes: (i) *x*−1/2, −*y*+1/2, −*z*+1; (ii) −*x*+3/2, *y*+1/2, *z*; (iii) −*x*+2, *y*−1/2, −*z*+3/2; (iv) *x*−1/2, *y*, −*z*+3/2; (v) −*x*+3/2, *y*−1/2, *z*; (vi) *x*+1/2, −*y*+1/2, −*z*+1; (vii) −*x*+2, *y*+1/2, −*z*+3/2; (viii) *x*+1/2, *y*, −*z*+3/2.

### Hydrogen-bond geometry (Å, °)

**Table:**

*D*—H···*A*	*D*—H	H···*A*	*D*···*A*	*D*—H···*A*
O13—H7···O8	0.93	1.73	2.609 (3)	158
